# Broadband Vibration Attenuation Achieved by 2D Elasto-Acoustic Metamaterial Plates with Rainbow Stepped Resonators

**DOI:** 10.3390/ma14174759

**Published:** 2021-08-24

**Authors:** Wenming Wei, Dimitrios Chronopoulos, Han Meng

**Affiliations:** 1 State Key Laboratory for Manufacturing Systems Engineering, School of Mechanical Engineering, Xi’an Jiaotong University, Xi’an 710049, China; wm_wei@xjtu.edu.cn; 2 Department of Mechanical Engineering & Division of Mechatronic System Dynamics (LMSD), KU Leuven, Ghent Technology Campus, 9000 Gent, Belgium; dimitrios.chronopoulos@kuleuven.be; 3 Department of Mechanical and Construction Engineering, Northumbria University, Newcastle NE1 8ST, UK

**Keywords:** stepped resonators, rainbow, metamaterial plates, vibration attenuation, broadband

## Abstract

This paper investigates the influences of nonperiodic rainbow resonators on the vibration attenuation of two-dimensional metamaterial plates. Rainbow metamaterial plates composed of thin host plates and nonperiodic stepped resonators are considered and compared with periodic metamaterial plates. The metamaterial plates are modelled with the finite element modelling method and verified by the plane wave expansion method. It was found that the rainbow metamaterial plates with spatially varying resonators possess broader vibration attenuation bands than the periodic metamaterial plate with the same host plates and total mass. The extension of attenuation bands was found not to be attributed to the extended bandgaps for the two-dimensional metamaterial plates, as is generally believed for a one-dimensional metamaterial beam. The complete local resonance bandgap of the metamaterial plates is separated to discrete bandgaps by the modes of nonperiodic resonators. Although the additional modes stop the formation of integrated bandgaps, the vibration of the plate is much smaller than that of resonators at these modal frequencies, the rainbow metamaterial plates could have a distinct vibration attenuation at these modal frequencies and achieve broader integrated attenuation bands as a result. The present paper could offer a new idea for the development of plate structures with broadband vibration attenuation by introducing non-periodicity.

## 1. Introduction

Metamaterials are artificial structures with extraordinary properties that cannot be found in naturally occurring materials. Metamaterials were first described in the area of electromagnetic wave control [1,2,3]. Many electromagnetic and optical metamaterials were proposed with unusual properties such as negative refractive index [4,5,6], negative permittivity [7,8], broadband absorption [9], etc. In the past two decades, the concept of metamaterials has been extended to the areas of acoustic and elastic wave control. The elasto-acoustic metamaterials have the ability of manipulating mechanical waves. Analogous to their counterparts in electromagnetics, the elasto-acoustic metamaterials also possess unique negative properties, such as negative effective mass/density [10,11,12,13], negative bulk modulus [14,15,16], double negativity [17,18,19,20], etc. The very first acoustic metamaterial, developed by Liu et al. [21], used fabricated sonic crystals with hard cores and soft coatings arranged in an epoxy matrix. Since then, the development of elasto-acoustic metamaterials has attracted considerable attention. For instance, many researchers (e.g., Sugino et al. [22], Pai et al. [23], Huang et al. [24], Liu et al. [25], Yu et al. [26], Xiao et al. [27,28] and Nobrega et al. [29]) have developed one-dimensional (1D) metamaterial rods and beams with single or multiple degree of freedom mass-spring resonators. Many others (e.g., Peng and Pai [30], Peng et al. [31], Miranda et al. [32], Wang et al. [33], He et al. [34], Zhu et al., Hsu [35], Zhang et al. [36], Qin et al. [37], Nouh et al. [38], Li et al. [39], Xiao et al. [40]) have investigated the vibration and noise control by two-dimensional (2D) metamaterial plates with a variety of attachments, such as a mass-spring resonator [30,31,32,33,34], stepped resonators [35,39], a built-in membrane with attached mass resonators [38], beam-like resonators [40], lumped masses [41,42], etc. Several studies (e.g., Elmadih et al. [43], Yuan et al. [44], Krushynska et al. [45,46], Jiang et al. [47], Mizukami et al. [48], An et al. [49], D’Alessandro [50], Poggetto et al. [51]) have proposed three-dimensional (3D) elasto-acoustic metamaterial lattices with local resonators composed of concentric hard and stiff inclusions or ligaments and connected mass bulks of different shapes in recent years.

One of the most important features of elasto-acoustic metamaterials is the existence of bandgaps, within which the propagation of waves is prohibited or greatly attenuated to required levels. The bandgaps can be generated by two mechanisms, Bragg scattering and local resonance. Bragg scattering bandgaps occur with *L = nλ/2*, where *L* is the structure periodicity dimension, and *λ* is the wavelength of the propagating waves. The occurrence of Bragg-type bandgaps requires the structure periodicity dimensions to be comparable to the wavelength of the propagating waves; therefore, it is difficult to achieve Bragg-type bandgaps at low frequencies. By contrast, the local resonance bandgaps are formed by the resonance of oscillators attached to or embedded in host structures. The periodicity dimensions of the structures could thus be much smaller than the wavelength of propagating waves.

The existence of local resonance bandgaps enables the metamaterials to have a great potential for the vibration attenuation at low frequencies. However, the widths of local resonance bandgaps are limited at low frequencies, which challenges the practical application of metamaterials. The elasto-acoustic metamaterials are originally periodic structures; “rainbow” metamaterials with nonperiodic units that can adjust the band structures were found to be capable of enhancing the vibration attenuation in recent research. Zhu et al. [52] and Wang [53] proposed rainbow trappings consisting of nonperiodic graded unit cells that could trap acoustic waves within broadband frequency ranges. Beli et al. [54] proved that the spatial correlated variability could lead to widened bandgaps for cantilever-in-mass metamaterials. Meng et al. [55,56,57,58,59] investigated rainbow metamaterial beams with spatially varying oscillators and rainbow phononic crystal lattices with spatial varying mass blocks and found that the rainbow structures could generate broader bandgaps compared with periodic ones.

The abovementioned rainbow structures were centered on 1D metamaterial beams or multi-dimensional phononic crystals. To the best of our knowledge, none of the existing papers have investigated the influences of nonperiodic rainbow resonators on 2D metamaterial plates. Plates are the fundamental elements of many engineering structures; metamaterial plates with effective broadband vibration attenuation have potential for applications with a demand for low-frequency vibration control, such as cabin walls, ceilings and floors of airplanes, trains and other vehicles, machines, etc. The development of plate structures with broadband low-frequency vibration attenuation could therefore be critical for both industry and academics.

Metamaterial plates with rainbow stepped resonators are first proposed in the present paper for the purpose of obtaining broader vibration attenuation bands. The masses of oscillators are spatially varying in two directions. The dispersion spectrums and frequency response functions (FRFs) of rainbow metamaterial plates are compared with those of a periodic structure of the same host plate and total mass. The mechanisms of the attenuation band extension are also revealed through the analysis of mode shapes. The present paper is structured as follow: Section 2 introduces the components of periodic and rainbow metamaterial plates and the finite element modelling (FEM) method. Section 3 introduces the validation of the FE models based on the plane wave expansion method. The influences of rainbow resonators on the dynamic properties of metamaterial plates are explored in Section 4.

## 2. FE Modelling of Metamaterial Plates

**Figure 1:**
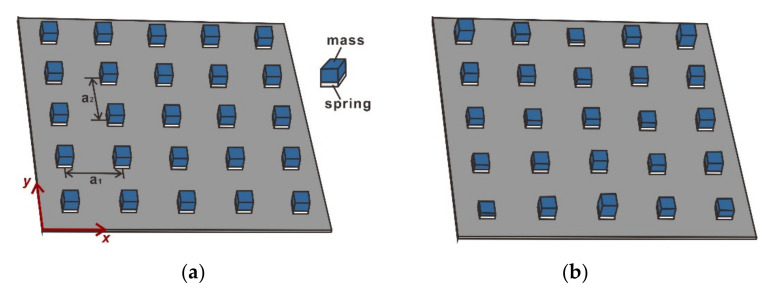
Schematic diagrams of metamaterial plates with (**a**) periodic and (**b**) rainbow stepped resonators. The distances between resonators in the x and y directions are $a_{1}$ and $a_{2}$, respectively.

Metamaterial plates with stepped resonators are considered in the present paper as shown in Figure 1a,b. The stepped resonators were composed of soft elastic materials as springs and hard blocks attached to the soft elastic materials as the masses. The stiffness and mass of the resonators could simply be adjusted by changing the dimensions and shapes of the soft and hard blocks. The resonators were periodically attached at the surface of the metamaterial plates, and distances between resonators in the x and y directions were $a_{1}$ and $a_{2}$, respectively. The periodic metamaterial plate contained identical stepped resonators (Figure 1a), whereas the rainbow metamaterial plate had stepped resonators of different masses (Figure 1b). It should be mentioned that the thickness of the host plate was much smaller than other dimensions and the flexural wavelength. The dimensions of the plates were assumed as $a_{1}=a_{2}=100\;\mathrm{mm}$, thickness h=2 mm, which is common for plate structures in the potential application areas. The material of the host plates and mass blocks was assumed to be aluminum with a Young’s modulus of e=70 Gpa, a density of $\rho =2700{\;\mathrm{kg}/m}^{3}$, and a Poisson’s ratio of ν=0.3. The flexural wavelength of the plate $\lambda =2\pi \sqrt[{4}]{Eh^{2}/12\left(1-\nu^{2}\right)\rho \omega^{2\;}}$ was thus also much larger than the thickness of the plate. The material of the springs was assumed to be rubber, with a Young’s modulus of $E_{s}=2.5\;\mathrm{Mpa}$, a density of $\rho_{s}=900{\;\mathrm{kg}/m}^{3}$, and a Poisson’s ratio of $\nu_{s}=0.45$.

Notably, it should be stressed that we focused on the flexural vibration of the metamaterial plate; only anti-symmetric (A mode) Lamb waves existed in the plate due to the thin layer assumption, and the resonators also oscillated in a normal direction to the plate with only the out-of-plane resonance modes being considered.

The dispersion spectrum and FRFs of the metamaterial plates were calculated by FE models. The host plates and stepped oscillators were treated as an integrated solid part modelled by the Solid Mechanics module of COMSOL Multiphysics. The plates and resonators were set as the Linear Elastic Material domains in the FE models.

## 3. Validation of the FE Models

The plane wave expansion method that can calculate the dispersion curves of periodic structures was employed in the present study to verify the FE models. The plane wave expansion method has been validated to effectively evaluate the band structures of metamaterials and phononic crystals [51,60,61].

Given that the thickness of the host plate was much smaller than its width and length, the displacement of the metamaterial plates and oscillating mass are given as [60]

(1) $$D{\left(\frac{\partial^{2}}{\partial x^{2}}+\frac{\partial^{2}}{\partial y^{2}}\right)}^{2}w_{1}\left(r\right)-\omega^{2}\rho ℎw_{1}\left(r\right)=\sum_{R}\left[f_{1}\left(R\right)\delta \left(r-R\right)\right]$$

(2) $$m_{r}{\ddot{w}}_{2}\left(R\right)=-f_{1}\left(R\right)$$

where $f_{1}\left(R\right)=-k_{S}\left(w_{1}\left(R\right)-w_{2}\left(R\right)\right)$, $m_{r}$ is the mass of the resonators, and $k_{S}$ is the stiffness of the springs, $w_{1}\left(r\right)$ and $w_{2}\left(R\right)$ are the displacements of the host plates and oscillators, respectively. $r=\left(x,y\right)$, $R=\left(ma_{1},na_{2}\right)$ represents the connection points of the oscillators at the host plate, m and n are integers. $D=Eh^{3}/12\left(1-\nu^{2}\right)$ is the bending stiffness of the plate.

Due to the periodicity of the metamaterial plate, the displacement of the plate can be written as [61],

(3) $$w_{1}\left(r\right)=\sum_{G}W_{1}\left(G\right)e^{-i\left(k+G\right)\cdot r}$$

where $k=\left(k_{x},k_{y}\right)$ denotes the wavenumbers, $k_{x}=k\sin\phi ,\;k_{y}=k\cos\phi$, ϕ is the azimuth angle of the vector, $G=\left(2\pi m/a_{1},2\pi n/a_{2}\right)$ denotes the reciprocal-lattice vector. The displacements of the host plate and oscillators can also be given as

(4) $$w_{1}\left(R\right)=w_{1}\left(0\right)e^{-ik\cdot R}$$

(5) $$w_{2}\left(R\right)=w_{2}\left(0\right)e^{-ik\cdot R}$$

Substitution of Equations (3)–(5) into Equations (1) and (2) yields,

(6) $$D{\left(\frac{\partial^{2}}{\partial x^{2}}+\frac{\partial^{2}}{\partial y^{2}}\right)}^{2}w_{1}\left(r\right)-\omega^{2}\rho ℎw_{1}\left(r\right)=-k_{r}\left(w_{1}\left(0\right)-w_{2}\left(0\right)\right)e^{-ik\cdot r}\sum \delta \left(r-R\right)$$

(7) $$-\omega^{2}m_{r}w_{2}\left(0\right)=k_{r}\left(w_{1}\left(0\right)-w_{2}\left(0\right)\right)$$

Since

(8) $$\sum_{R}\delta \left(r-R\right)=\sum_{G}\tilde{g}\left(G\right)e^{-iG\cdot r}=\frac{1}{S}\sum_{G}e^{-iG\cdot r}$$

where $\tilde{g}\left(G\right)=\frac{1}{S}\iint_{S}\left(\sum_{r}\delta \left(r-R\right)\right)e^{iG\cdot r}d^{2}r=\frac{1}{S}$, $S=a_{1}a_{2}$.

Substitution of Equations (8) and (7) into Equation (6) yields,

(9) $$\begin{array}{c} DS{\left({\left(k_{x}+m\frac{2\pi }{a_{1}}\right)}^{2}+{\left(k_{y}+n\frac{2\pi }{a_{2}}\right)}^{2}\right)}^{2}W_{1}\left(G\right)-S\omega^{2}\rho hW_{1}\left(G\right)\\ =\frac{k_{r}\omega^{2}m_{r}}{k_{r}-\omega^{2}m_{r}}\sum_{G}W_{1}\left(G\right) \end{array}$$

To solve the above equation, the infinite summation needs to be truncated. Assuming m,n=−M,…,0,…M, the above equation could then be rewritten as,

(10) $$\left[\left(DSK\right)-S\rho h\omega^{2}I+D_{R}U\right]W_{1}=0$$

where

(11) $$\begin{array}{c} \begin{array}{c} U={\left[\begin{array}{ccc} 1 & \ldots & 1\\ \vdots & \ddots & \vdots \\ 1 & \cdots & 1 \end{array}\right]}_{{\left(2M+1\right)}^{2}\times {\left(2M+1\right)}^{2}} \end{array}\\ I=\mathrm{diag}\left({\left[1,1,\ldots ,1\right]}_{1\times {\left(2M+1\right)}^{2}}\right)\\ v_{m,n}={\left({\left(K_{x}+\frac{2\pi m}{a_{1}}\right)}^{2}+{\left(k_{y}+\frac{2\pi n}{a_{2}}\right)}^{2}\right)}^{2}\\ K=\mathrm{diag}\left({\left[v_{\left(-M\right),\left(-M\right)},v_{\left(-M+1\right),\left(-M\right)},\ldots ,v_{0,0},\ldots ,v_{M,M}\right]}_{1\times {\left(2M+1\right)}^{2}}\right) \end{array}$$

The wavenumbers could be solved with a given angular frequency and azimuth angle. The band structures of the periodic metamaterial plate can thus be obtained with the calculated wavenumbers.

**Figure 2:**
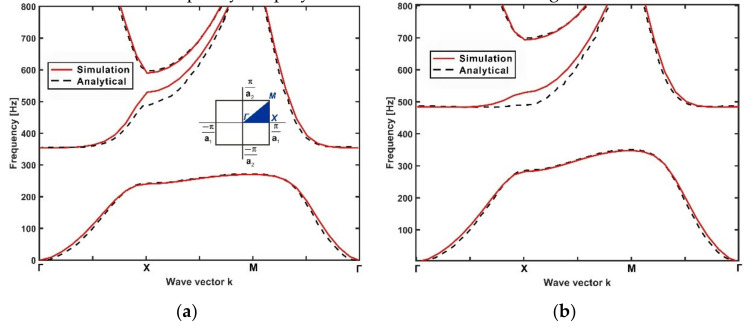
Comparison between the dispersion spectrum of metamaterial plates calculated by FEM (solid line) and the analytical model (dashed line). The Brillouin zone of the metamaterial plate is shown in the subfigure. The geometric parameters of the host plates were: $a_{1}=a_{2}=100\;\mathrm{mm}$, h=2 mm. The mass of the resonators was $m_{R}=0.027\;\mathrm{kg}$. The stiffnesses of the springs were (**a**) $k_{S}=93950\;N/m$ and (**b**) $k_{S}=192500\;N/m$.

The dispersion curves of two periodic metamaterial plates estimated by the FE models are compared with that obtained by the analytical model in Figure 2a,b. The dimensions and materials of the metamaterial plates were the same as mentioned in Section 2. The stiffnesses of the springs attached to the two plates were $k_{S}=93950\;N/m$ and $k_{S}=192500\;N/m$, respectively.

It can be seen from Figure 2a,b that the FE simulation agrees well with the analytical model for both metamaterial plates. The minor discrepancies could be attributed to the simplifications and calculation errors introduced by both the analytical model and FE models, such as the ideal thin plate assumption of the host plate and the ideal point connection between the host plate and resonators in the analytical model, the influences of mesh quality, employed solvers in the FE modelling method, etc.

## 4. Results and Discussion

In order to exhibit the influences of the rainbow resonators and reveal the underlying mechanisms, the dynamic properties of periodic and rainbow metamaterial plates with the same host plates, springs and total resonator mass are calculated and compared in this section. The materials and unit dimensions of the metamaterial plates were the same as that mentioned in Section 2. The springs of the resonators had a stiffness of 21,033 N/m. The mass of the resonators was assumed as 30% of that of the host plate. It should be noted that the bandgap frequency decreased with the increase in resonator mass, and the resonator mass could be adjusted according to the requirement of applications. The number of the units were both eight in the x and y directions, and the metamaterial plate thus contained 64 resonators in total.

Two rainbow metamaterial plates with linearly and sinusoidally varying resonator masses are considered in this section. Linear and sinusoidal distributions were employed as they are the most common nonperiodic distributions and other more complex distributions can easily be generated based on the piles of these two distributions [58]. It should be stressed that the presented resonator distributions are not optimal; they were selected to explore the effects of a rainbow design, and optimization of the rainbow resonator distributions will be the next step in our work.

**Figure 3:**
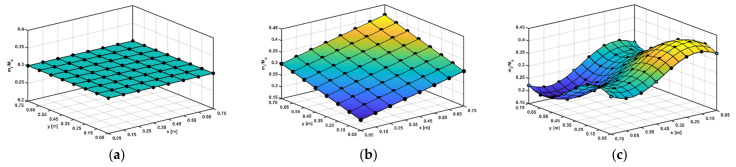
Resonator mass distributions of metamaterial plates with (**a**) periodic, (**b**) linearly varying and (**c**) sinusoidally varying resonators. $M_{a}$ is a unit mass of the host plate and $m_{r}$ is the mass of the resonator attached to the unit of the metamaterial plates.

The two rainbow metamaterial plates with linearly varying and sinusoidally varying resonators have resonator mass distributions as

(12) $$\frac{m_{r}}{M_{a}}=0.175x+0.175y+0.21$$

(13) $$m_{r}/M_{a}=0.07\sin\left(\frac{20}{9}\pi x+\frac{\pi }{9}\right)+0.07\sin\left(\frac{20}{9}\pi y+\frac{\pi }{9}\right)+0.35$$

where $M_{a}$ is a unit mass of the host plate and $m_{r}$ is the mass of the resonator attached to a unit of the metamaterial plate. The resonator mass distributions of the periodic and rainbow metamaterial plates are depicted in Figure 3a–c.

Supposing that the finite metamaterial plates are subjected to an excitation force at one point of the host plates, the transmissibility values $T_{r}$ could be achieved by the ratios between the displacements at the excitation and sampling points:

(14) $$T_{r}\left(f\right)=20\log_{10}\left|\frac{u_{s}}{u_{e}}\right|$$

where $u_{e}$ and $u_{s}$ are the evaluated displacements at the excitation and sampling points, respectively. The metamaterial plates were assumed as being subjected to free boundary conditions unless otherwise noted.

### 4.1. Periodic Metamaterial Plate

**Figure 4:**
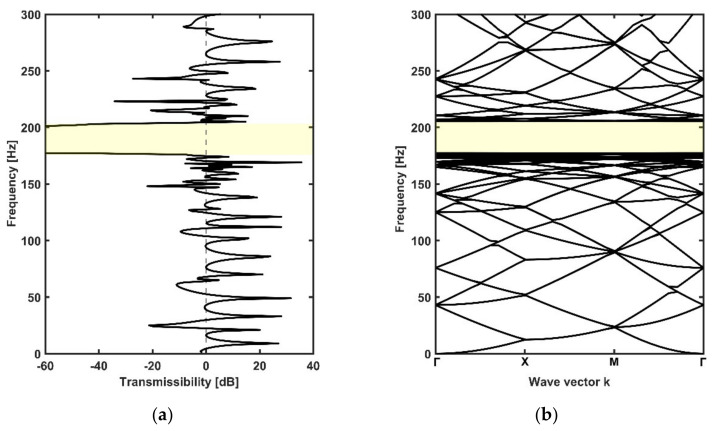
The transmissibility (**a**) and dispersion spectrum (**b**) of the periodic metamaterial plate. The bandgap region of 175–204 Hz is marked in yellow.

The transmissibility values and dispersion curves of the periodic metamaterial plate are shown in Figure 4a,b. It should be noted that the dispersion curves of finite rainbow metamaterial plates needed to be calculated by assuming that the finite plates were the unit cells of infinite metamaterial plates. In order to have a better comparison with the rainbow metamaterials, the dispersion curves of periodic metamaterial plates were obtained with the whole finite periodic plate that had the same area and number of resonators as the rainbow metamaterials instead of a single unit. It can be seen from the two figures that a complete bandgap took place within the frequency range of 175–204 Hz. This bandgap was obviously caused by local resonance because the bandgap frequency matched with the resonance frequency (i.e., 181 Hz) of the periodic stepped resonators.

**Figure 5:**
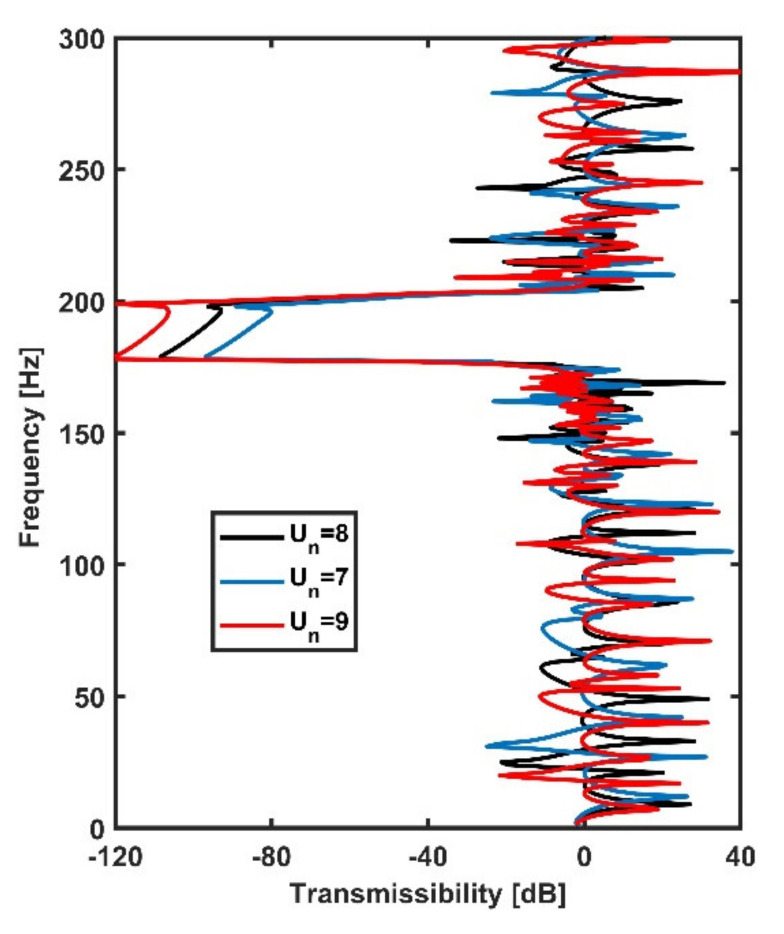
The comparison of transmissibility of metamaterial plates with different numbers of resonators. $U_{n}$ denotes the number of units in the x and y directions. The blue, black and red lines represent plates with $U_{n}=7,\;8,\;9$, respectively.

The influences of resonator numbers on the transmissibility of the plate are explored in Figure 5. The transmissibility values of three periodic metamaterial plates with $U_{n}=7,\;8,\;9$ are compared, where $U_{n}$ denotes the number of units in the x and y directions. It can be seen from Figure 5 that increasing the number of resonators and plate dimensions could enhance the attenuation values but could not change the bandgap frequency and bandwidth.

**Figure 6:**
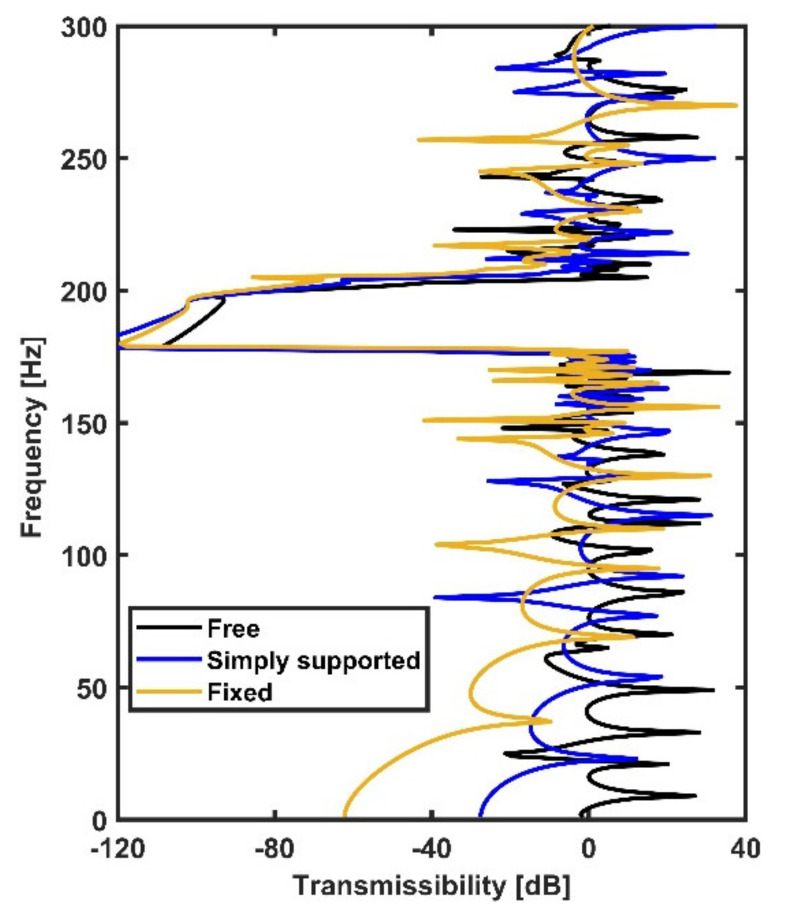
The comparison of transmissibility of metamaterial plates subjected to different boundary conditions: free boundaries (black line); fixed boundaries (yellow line); and simply supported boundaries (blue line).

As is known, the dynamic properties of plate structures are greatly influenced by their boundary conditions. The influences of plate boundary conditions on the transmissibility of the metamaterial plates are also revealed in this section. Figure 6 compares the transmissibility of metamaterial plates with various common boundary conditions (i.e., free, fixed and simply supported boundaries). It was found that different boundary conditions can generate different transmissibility values; metamaterial plates with fixed and simply supported boundaries showed better performance at low frequencies compared with the plate subjected to free boundaries. However, the bandgap frequency and bandwidth were slightly affected by the boundary conditions.

### 4.2. Rainbow Metamaterial Plate

**Figure 7:**
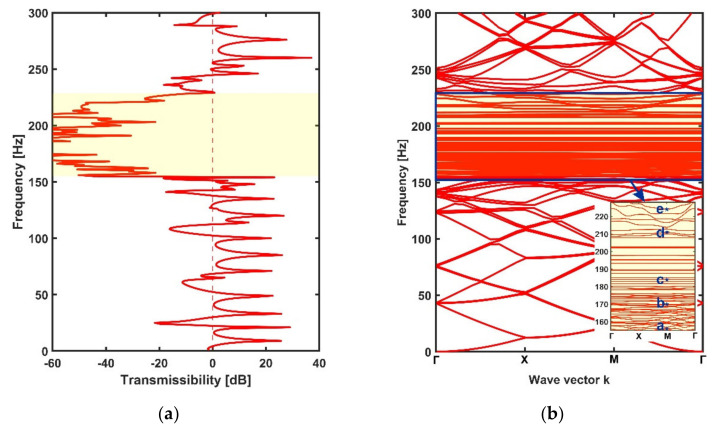
The transmissibility (**a**) and dispersion spectrum (**b**) of the rainbow metamaterial plate with linearly varying resonators. The attenuation band of 155–228 Hz is marked in yellow. The dispersion curves within the attenuation band are shown in the subfigure of (**b**).

**Figure 8:**
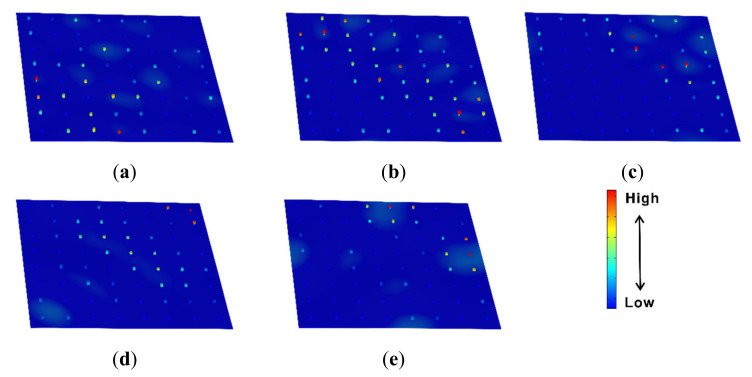
Mode shapes of the rainbow metamaterial plate with linearly varying resonators at the points (**a**) a=155.9 Hz, (**b**) b=171.7 Hz, (**c**) c=185.1 Hz, (**d**) d=212.8 Hz and (**e**) e=224.1 Hz as marked in Figure 7b.

The transmissibility values and dispersion curves of the rainbow metamaterial plate with linearly varying resonators are shown in Figure 7a,b, respectively. It can be seen from Figure 7a that the rainbow metamaterial plate exhibits great vibration reduction in the frequency range of 155–228 Hz, which is 2.5 times wider than the bandgap of the periodic metamaterial plate. However, in contrast, the dispersion curves do not possess an integrated bandgap consistent with the vibration attenuation band (i.e., the spectrum band of 155–228 Hz within which considerable attenuation could be achieved) as shown in Figure 7b. The 2D metamaterial plates were hence different from the 1D metamaterial beams that showed extended attenuation bands as well as bandgaps due to the existence of rainbow resonators [56]. The whole bandgap of the periodic metamaterial plate was replaced by narrower completed and directional bandgaps owing to the modes of the distributed nonperiodic resonators as shown in the subfigure of Figure 7b. The mode shapes at the points a =155.9 Hz, b=171.7 Hz, c=185.1 Hz, d=212.8 Hz and e=224.1 Hz in the dispersion curves within the attenuation band are shown in Figure 8a–e. It can be seen from these mode shape figures that the host plate and resonators vibrated simultaneously at these modal frequencies, but the resonators apparently had a much larger vibration amplitude than that of the host plate, which means that the vibration of the plate can still be reduced by the resonators at these modal frequencies. Thus, the rainbow metamaterial plate had effective vibration attenuation within the whole attenuation band region. In addition, compared with the periodic metamaterial plate, the rainbow metamaterial plate with linearly varying resonators had varied resonator masses, which then had varied resonance frequencies ranging from 156 Hz to 225 Hz, and the attenuation band region was therefore much wider than that of the periodic metamaterial plate.

**Figure 9:**
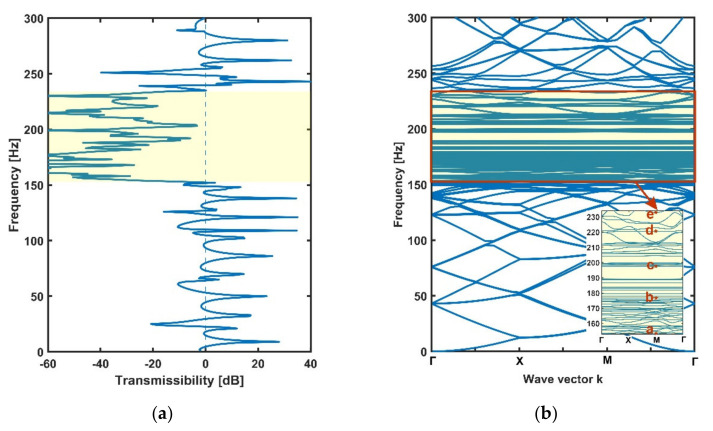
The transmissibility (**a**) and dispersion spectrum (**b**) of the rainbow metamaterial plate with sinusoidally varying resonators. The attenuation band of 153–234 Hz is marked in yellow. The dispersion curves within the attenuation band are shown in the subfigure of (**b**).

**Figure 10:**
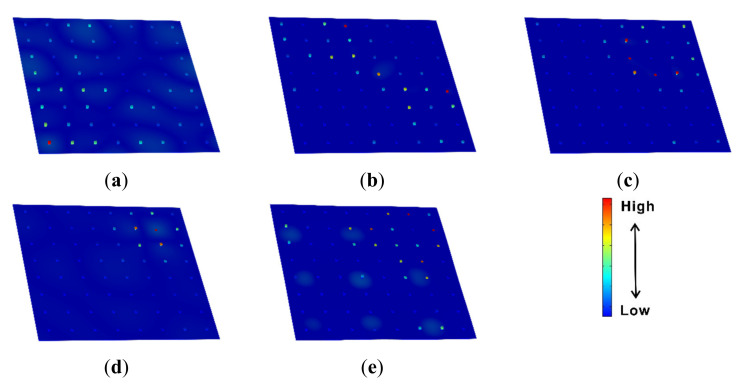
Mode shapes of the rainbow metamaterial plate with sinusoidally varying resonators at the points (**a**) a=153.3 Hz, (**b**) b=176.6 Hz, (**c**) c=197.6 Hz, (**d**) d=220.5 Hz and (**e**) e=232.7 Hz as marked in Figure 9b.

Similar findings could be obtained based on the dispersion spectrum and transmissibility of the rainbow metamaterial plate with sinusoidally varying resonators as shown in Figure 9a,b. The considered rainbow metamaterial plate had an extended attenuation band compared with the periodic metamaterial as shown in Figure 9a. The mode shapes at the points a=153.3 Hz, b=176.6 Hz, c=197.6 Hz, d=220.5 Hz and e=232.7 Hz on the dispersion curves shown in the subfigure of Figure 9b are displayed in Figure 10a–e. It can also be concluded that although the modes of the nonperiodic resonators could truncate the complete bandgap, the attenuation band could be broadened owing to the varied resonance frequencies of these nonperiodic resonators.

## 5. Conclusions

The vibration attenuation of 2D rainbow metamaterial plates with spatially varying stepped resonators was investigated in the present paper. By comparing the dispersion spectrum and transmissibility of the two rainbow metamaterial plates with those of the periodic metamaterial plate, it was found that rainbow resonators could lead to wider vibration attenuation bands compared with periodic resonators. Although the additional mode shapes of the rainbow resonators could break the complete bandgap of the periodic metamaterial plate in to isolated narrower bandgaps, the vibration amplitude of the host plates was much smaller compared with that of the vibrating resonators at these modal frequencies, i.e., the vibration of the host plates was still largely reduced by the resonators. Extended integrated vibration attenuation bands were therefore formed regardless of the separated narrower bandgaps.

The idea of broadening the attenuation band by non-periodicity proposed in the present paper could be instructive for future researchers to design more plate structures with better vibration attenuation. The investigated plane single-layered metamaterial plate could also be easily extended to plane and curved single-layered or multilayered plate structures for wider applications.

## Data Availability

The data presented in this study are contained within the article.
